# Cellular calcification induced by inorganic polyphosphate involves ATP depletion and opening of the mitochondrial permeability transition pore (mPTP)

**DOI:** 10.1002/2211-5463.12703

**Published:** 2019-07-30

**Authors:** Kaori Tsutsumi, Tatsuya Sasase

**Affiliations:** ^1^ Faculty of Health Sciences Hokkaido University Sapporo Japan; ^2^ Graduate School of Health Sciences Hokkaido University Sapporo Japan

**Keywords:** adenosine triphosphate, calcification, inorganic polyphosphate, mitochondria, mitochondrial permeability transition pore

## Abstract

Inorganic polyphosphate (polyP) is a linear polymer containing tens to hundreds of orthophosphate residues linked by high‐energy phosphoanhydride bonds. PolyP promotes osteocalcification and bone mineralization in both mouse and human osteoblastic cells. In the present study, we examined the molecular mechanism by which polyP affects mitochondrial metabolism to promote cellular calcification in MC3T3‐E1 osteoblastic cells. The cellular content of adenosine triphosphate (ATP) was diminished one day after polyP treatment, and this was accompanied by increased conversion to adenosine diphosphate. Furthermore, mitochondrial membrane potential was significantly decreased in polyP‐treated cells. These results suggest that the depletion of intracellular ATP and the decrease in mitochondrial membrane potential induced by polyP treatment may be a trigger to promote cell calcification.

**Abbreviations**
ADP: adenosine diphosphate
ATP: adenosine triphosphate
mPTP: mitochondrial permeability transition pore
polyP: inorganic polyphosphate

Inorganic polyphosphate (polyP) is a linear polymer containing tens to hundreds of orthophosphate residues linked by high‐energy phosphoanhydride bonds; it is found in all living organisms from bacteria to mammals 1, 2. Recently, it has been found that polyP has many important physiological functions in mammalian cells 1, 2, 3, 4, 5, 6.

The PolyP promotes osteocalcification and bone mineralization in both mouse and human osteoblastic cells 7, 8, 9, 10. Regarding the effect of polyP on cell calcification, Kawazoe *et al*. 9 reported that it promotes cell calcification in murine osteoblastic MC3T3‐E1 cells, while Morita *et al*. 11 reported that inorganic polyphosphate‐adsorbed hydroxyapatite enhances bone regeneration, and cellular calcification of MC3T3‐E1 cells is enhanced on the surface of polyP‐adsorbed hydroxyapatite 12. Kawazoe also reported that in the calcification of MC3T3‐E1 cells, polyP is utilized as a phosphate source and that this is accompanied by an increase in polyphosphatase activity 9. Additionally, Müller *et al*. 13, 14 applied an amorphous polyphosphate–hydroxyapatite study in human osteogenic sarcoma cells (SaOS‐2). Furthermore, Wang *et al*. suggested the occurrence of dual effects of polyP; that is, polyP with Ca^2+^ regulates bone metabolism via dual effects of hydroxyapatite producing in SaOS‐2 cells and hydroxyapatite resorbing in the murine monocyte/macrophage cell line (RAW 264.7 cells). Wang *et al*. also reported that initial mineralization is modulated by carbonic anhydrase activators and polyphosphate in SaOS‐2 15 and polyP induces expression of the Ets1 gene and alkaline phosphatase in SaOS‐2 16.

The PolyP also plays critical role in mitochondrial function by regulating mitochondrial energy metabolism 17. Further, polyP was proposed to play a crucial role in the activation of the mitochondrial permeability transition pore (mPTP) via calcium‐induced formation of the complex with C‐subunit and PHB 18, 19, 20. mPTP is a large, weakly selective channel present in the mitochondrial inner membrane; it plays an important role in controlling the mitochondrial membrane potential, the mitochondrial content of adenosine triphosphate (ATP), and the uptake and efflux of calcium ions between mitochondria and cytoplasm by mPTP opening 21, 22. mPTP activation leads to mitochondrial swelling and rupture, and cell death 23.

In our previous study, using electron microscopy, we observed that polyP treatment causes mitochondrial elongation and swelling in polyP‐treated MC3T3‐E1 cells. In the present study, we further investigate effects of polyP on cell bioenergetics and mitochondrial function.

## Materials and methods

### Cell culture and reagents

MC3T3‐E1 cells (Riken Cell Bank, Tokyo, Japan) were cultured in α‐minimum essential medium (α‐MEM; Sigma‐Aldrich Co., St. Louis, MO, USA) supplemented with 10% fetal bovine serum (FBS; Cansera International Inc., Etobicoke, Ontario, Canada), and 100 units·mL^−1^ penicillin and 100 μg·mL^−1^ streptomycin (Sigma‐Aldrich Co., St. Louis, MO, USA) at 37 °C in a humidified atmosphere containing 5% CO_2_. The culture medium was changed every 3 days. PolyP chains of all lengths were kindly provided by Dr. Shiba (RegeneTiss Inc., Tokyo, Japan). As a control for polyP treatment, the culture medium mentioned above was used without additive (None) and with sodium phosphate buffer (pH 6.9) (Na‐phosphate).

### Alizarin red S staining

After the cells had been cultured to confluence in a six‐well dish, the cell culture medium was changed to medium supplemented with 0.5% FBS. After 24 h, the medium of the cells was changed again to a medium with 0.5% FBS, 0.5% FBS and 1 mm sodium phosphate, or 0.5% FBS and 1 mm polyP, followed by culturing for 40 days. The culture medium was changed every 3 days, maintaining the same composition. The cells were washed with phosphate‐buffered saline (PBS) three times and fixed with ice‐cold methanol for 20 min at 4 °C. The fixed cells were washed again with distilled water once and stained with Calcified Nodule Staining kit (Cosmo Bio Co., Ltd., Tokyo, Japan).

### Cell growth

To compare cell growth between control cells and polyP‐treated cells, 5000 cells were cultured on each well of 96‐well plates for 24 h, after which the culture medium was replaced by medium supplemented with 0.5% FBS. After 24 h, the cells were treated with 1 mm sodium phosphate (pH 6.8) or 1 mm polyP, and then, the cell growth was monitored by an MTS assay (CellTiter 96^®^ AQueous One Solution Cell Proliferation Assay; Promega Corporation, Madison, WI, USA). The absorbance at 490 nm was measured with a Model 680 Microplate Reader ( Bio‐Rad Laboratories, Inc., Hercules, CA, USA).

### Mitochondrial membrane potential assay

For the mitochondrial membrane potential assay, 5000 cells were cultured on each well of 96‐well plates for 24 h, after which the culture medium was replaced with medium supplemented with 0.5% FBS. After 24 h, the cells were treated with 1 mm sodium phosphate (pH 6.8) or 1 mm polyP for 48 h. The culture medium was removed and replaced with Cell Meter^™^ JC‐10 (AAT Bioquest, Inc., Sunnyvale, CA, USA) in PBS. After the cells had been incubated at 37 °C in 5% CO_2_ for 30 min, fluorescent intensities (Ex/Em = 490 nm/535 nm) were detected by AVRO MX (PerkinElmer, Inc., MA, USA). The ratio of mitochondrial membrane potential was estimated based on that at 0 h.

### Adenosine triphosphate assay

For the adenosine triphosphate (ATP) assay, cells were seeded in a 96‐well plate at a density of 5000 cells/well for 16 h, after which the cell medium was replaced by a fresh cell culture containing 0.5% FBS. After a further 24 h, the cells were treated with 1 mm sodium phosphate (pH 6.8) or 1 mm polyP. The changes of cellular ATP contents in each well were measured using CellTiter‐Glo^®^ 2.0 Assay kit (Promega Corporation) at the time indicated. The chemical luminescence was detected by AVRO MX (PerkinElmer, Inc.).

### Adenosine diphosphate/adenosine triphosphate ratio

To determine the adenosine diphosphate (ADP)/ATP ratio, the cells were seeded into a 96‐well plate at a density of 5000 cells/well for 16 h, after which the cell medium was replaced by a fresh cell culture containing 0.5% FBS. After a further 24 h, the cells were treated with 1 mm sodium phosphate (pH 6.8) or 1 mm polyP. The changes of cellular ATP contents in each well were measured using EnzyLight^™^ ADP/ATP Ratio Assay Kit (BioAssay Systems, Hayward, CA, USA) at the time indicated. The chemical luminescence was detected by AVRO MX (PerkinElmer, Inc., Waltham, MA, USA).

### Statistical analysis

All data from experiments using 96‐well plates were analyzed using spss version 18.0 (SPSS Inc., Chicago, IL, USA). Comparison among the three groups was performed by one‐way ANOVA and Tukey HSD test or Kruskal–Wallis test. Error bars represent the standard deviations (±SD) for the descriptive statistics.

## Results and Discussion

### Effect of chain length of polyP on calcification

**Figure 1:**
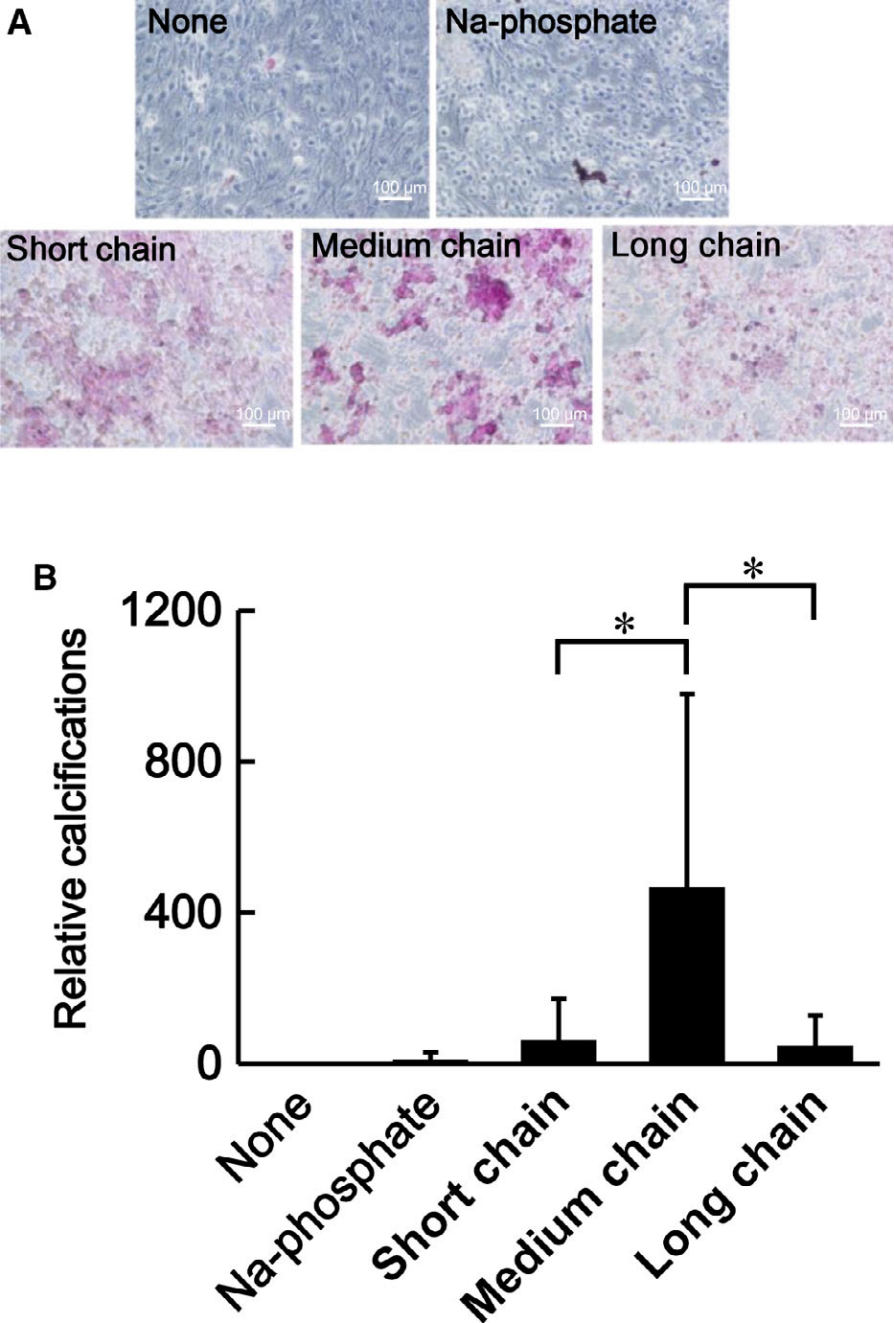
Effect of chain length of polyP on calcification. Calcification was visualized with Alizarin red S staining in polyP‐treated cells. The cells were treated with polyP of different chain lengths: short chain, average chain length of 14; medium chain, average chain length of 60; and long chain: average chain length of 130. (A) The original images of Alizarin red S staining. Scale bar means 100 μm. (B) The relative calcifications based on nontreated cells were quantitated by ImageJ, and average values of 6 different fields are shown with standard deviations. *P < 0.05. Comparison of five groups was performed by Kruskal–Wallis test.

We have reported that polyP potentially has the ability to promote cellular calcification 8, 9. In the present study, we first determined the most effective chain length of polyP for cell calcification. We used three different polyP lengths: short chain, medium chain, and long chain. The average chain lengths of these polyP types were 14, 60, and 120, respectively. Figure 1 indicates that the medium chain length was the most effective chain length for polyP to induce calcification in MC3T3‐E1 cells (Fig. 1A,B). As such, we used medium‐chain‐length polyP in subsequent experiments.

### Cell growth after polyP treatment

**Figure 2:**
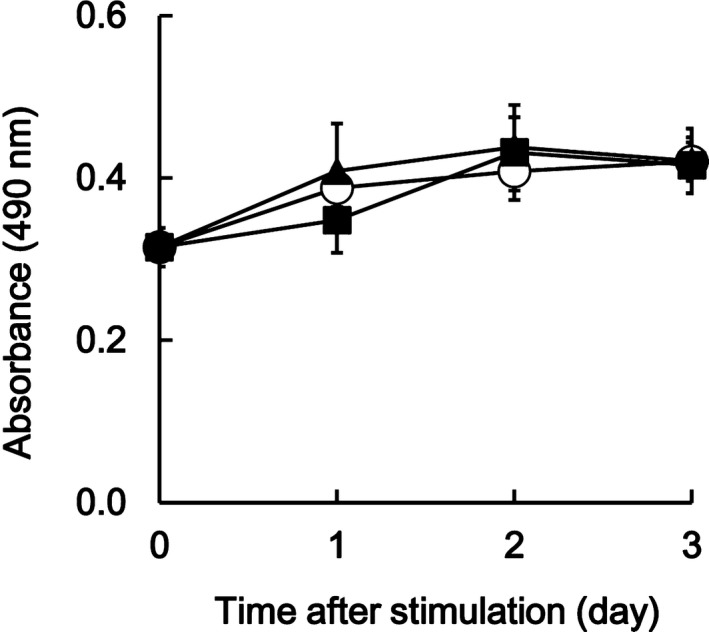
Cell growth. The cell growth after treatment with polyP. The growth of the cells after treatment with polyP in comparison with that for untreated and sodium phosphate‐treated groups. The cell growth was monitored by an MTS assay. ▲ stands for 1 mm sodium phosphate (pH 6.8), ■ for 1 mm polyP, and ○ for the untreated case. The plots represent the mean (±SD) of sextuplicate experiments.

We next investigated the effects of polyP treatment on cell growth. Cell growth was monitored by MTS assay. Although all groups were under 0.5% FBS conditions, they did not exhibit exponential growth; the MTS assay showed that polyP did not affect cell growth during 3 days after stimulation, when compared with the level in the control groups (Fig. 2). The reason why all groups did not grow significantly is that the culture medium contained only 0.5% FBS, with the condition to promote cellular calcification.

### PolyP lowers mitochondrial membrane potential

**Figure 3:**
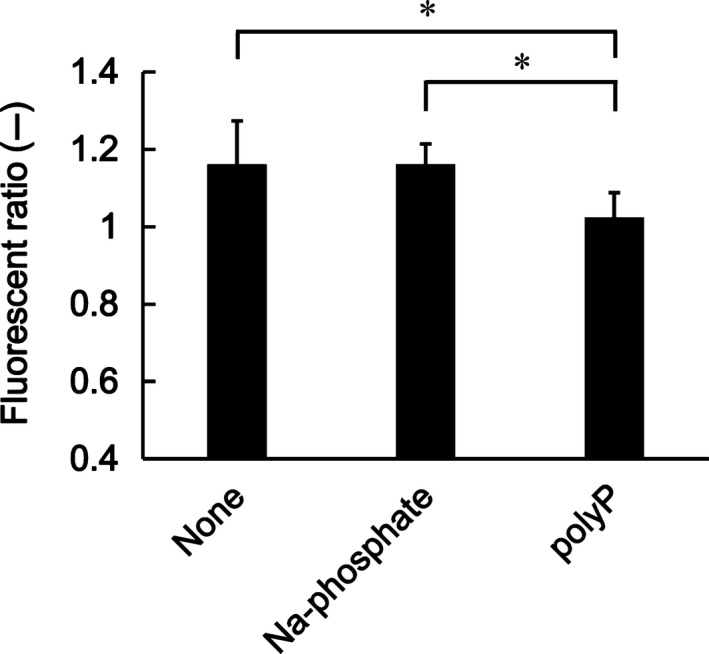
Mitochondrial potential. The mitochondrial potential was measured using Cell Meter^™^ JC‐10 (AAT Bioquest, Inc., CA, USA). The values represent the mean (±SD) of sextuplicate experiments. *P < 0.05 versus controls. *P* values were determined by one‐way ANOVA and Tukey HSD test.

In our previous study, we used electron microscopy to show that the mitochondria in polyP‐treated MC3T3‐E1 cells were elongated and swollen (Fig. S1) 9. As polyP acts as a potent activator and maintains mPTP opening 19, 24, 25, the treatment of osteoblastic‐like cells with polyP might lead to the activation of mPTP opening. Here, we measured the mitochondrial membrane potential 48 h after polyP treatment in Fig. 3, as revealed by fluorescent intensity after cell staining with Cell Meter^™^ JC‐10, an indicator dye whose intensity depends on mitochondrial potential. The mitochondrial membrane potential was significantly diminished in polyP‐treated cells compared with that in the control groups (None and Na‐phosphate) (Fig. 3). These results suggest that polyP introduces a decrease in mitochondrial potential, and it seems to be a cause of mitochondrial swelling in polyP‐treated cells. It was considered that this decreased mitochondrial potential by polyP is the membrane depolarization within a range that did not cause necrosis with considering the results of Fig. 2.

### Effect of polyP on nucleotide metabolism

**Figure 4:**
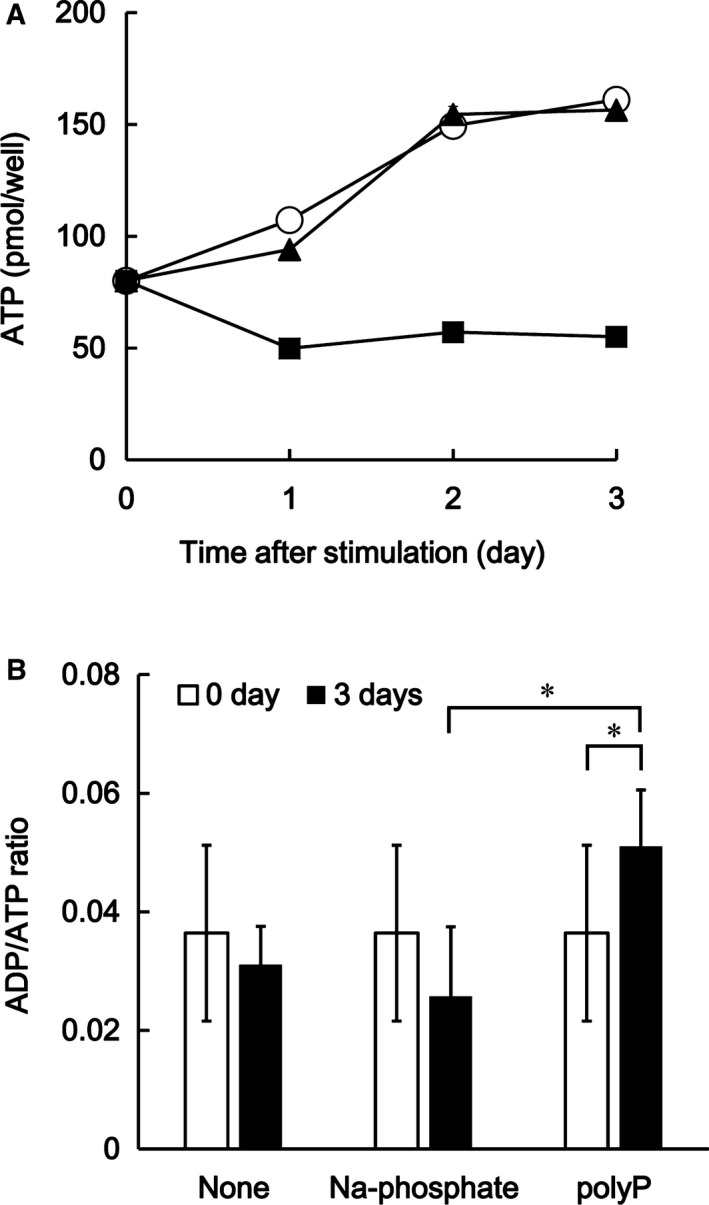
The change of cellular content of the adenosine triphosphate. (A) The cellular content of ATP was measured by CellTiter‐Glo^®^ 2.0 Assay kit (Promega Corporation, USA). The ▲ stands for 1 mm sodium phosphate (pH 6.8), ■ for 1 mm polyP, and ○ for untreated cells. The values represent the mean (± SD) of sextuplicate experiments. (B) The ADP/ATP ratio in the cells was measured between the two control groups and polyP‐treated cells using EnzyLight^™^ ADP/ATP Ratio Assay Kit (BioAssay Systems, CA, USA). The open bar and closed bar indicate 0 days and 3 days after treatment, respectively. The values represent the mean (±SD) of sextuplicate experiments. *P < 0.05. *P* values were determined by one‐way ANOVA and Tukey HSD test.

Since Kawazoe *et al*. reported that the levels of polyPase activity were enhanced in polyP‐treated MC3T3‐E1 cells 9, it was considered that dissociated orthophosphate would become the source of nucleotides such as ATP. On the other hand, mPTP opening results in mitochondrial swelling accompanied by ATP depletion 21. Thus, we next measured the change of cellular contents of ATP in polyP‐treated cells. Surprisingly, the cellular content of ATP was decreased in polyP although cell growth increased over time (Fig. 4A). The differences in cellular ATP contents between control groups and polyP‐treated cells were approximately threefold. In accordance with the mitochondrial swelling in polyP‐treated cells, it was thought that the depletion of ATP in polyP cells might result in the mPTP opening by polyP, which was a trigger to promote cell calcification. However, Müller *et al*. 13 reported that polyP increases the number of mitochondria and the ATP content within osteoblast‐like cells in SaOS‐2 cells after exposure to polyP. In contrast, the present report showed the ATP disruption in polyP‐treated cells. These two results are directly contradictory. Müller *et al*. 13 also mentioned in their report that the initial content of ATP might control the effect of polyP on the cellular content of ATP, they also asserted that amorphous Ca^2+^ polyphosphate nanoparticles regulate the ATP level in bone‐like SaOS‐2 cells 13. Therefore, we also suppose the possibility that polyP regulates the cellular contents of ATP depending on the cellular conditions, such as initial ATP and Ca^2+^ concentration, and controls the balance of cellular calcification by influencing mitochondrial metabolism. To initiate the promotion of cellular calcification, it might be important to stop energy production.

We next measured the ADP/ATP ratio in the cells and compared this between the two control groups and polyP‐treated cells. The results showed that the ADP/ATP ratio in the cells of control groups did not differ between before and after the treatment (Fig. 4A). On the other hand, the ADP/ATP ratio was significantly increased in polyP‐treated cells (Fig. 4B). These results suggest that ATP depletion in polyP‐treated cells might result in the conversion of ATP to ADP or the inhibition of ATP synthesis by polyP treatment.

## Conclusions

The depletion of intracellular ATP and the decrease in mitochondrial membrane potential by mitochondrial permeability transition pore opening appear to trigger cell calcification by polyP treatment. Further study of this is necessary, but polyP might promote cellular calcification via regulating cellular ATP levels while affecting mitochondrial metabolism.

## Conflict of interest

The authors declare no conflict of interest.

## Author contributions

KT designed the study. KT and TS performed the experiments, and KT wrote the manuscript. All authors reviewed the manuscript.

## Supporting information


**Fig. S1.** Electron micrograph of the cells. A. PolyP‐treated cells cultured on Biocoat Control Insert Micron (Becton Dickinson Ltd., USA) were observed using a transmission electron microscope (H‐800; Hitachi, Tokyo, Japan). Scale bar means 1 μm.
