# Supplementary figures and images for: Cellular calcification induced by inorganic polyphosphate involves ATP depletion and opening of the mitochondrial permeability transition pore (mPTP)
Source: FEBS Open Bio. 2019 Jul 30;9(9):1617–22. doi: 10.1002/2211-5463.12703 (PMC6722881; doi:10.1002/2211-5463.12703)

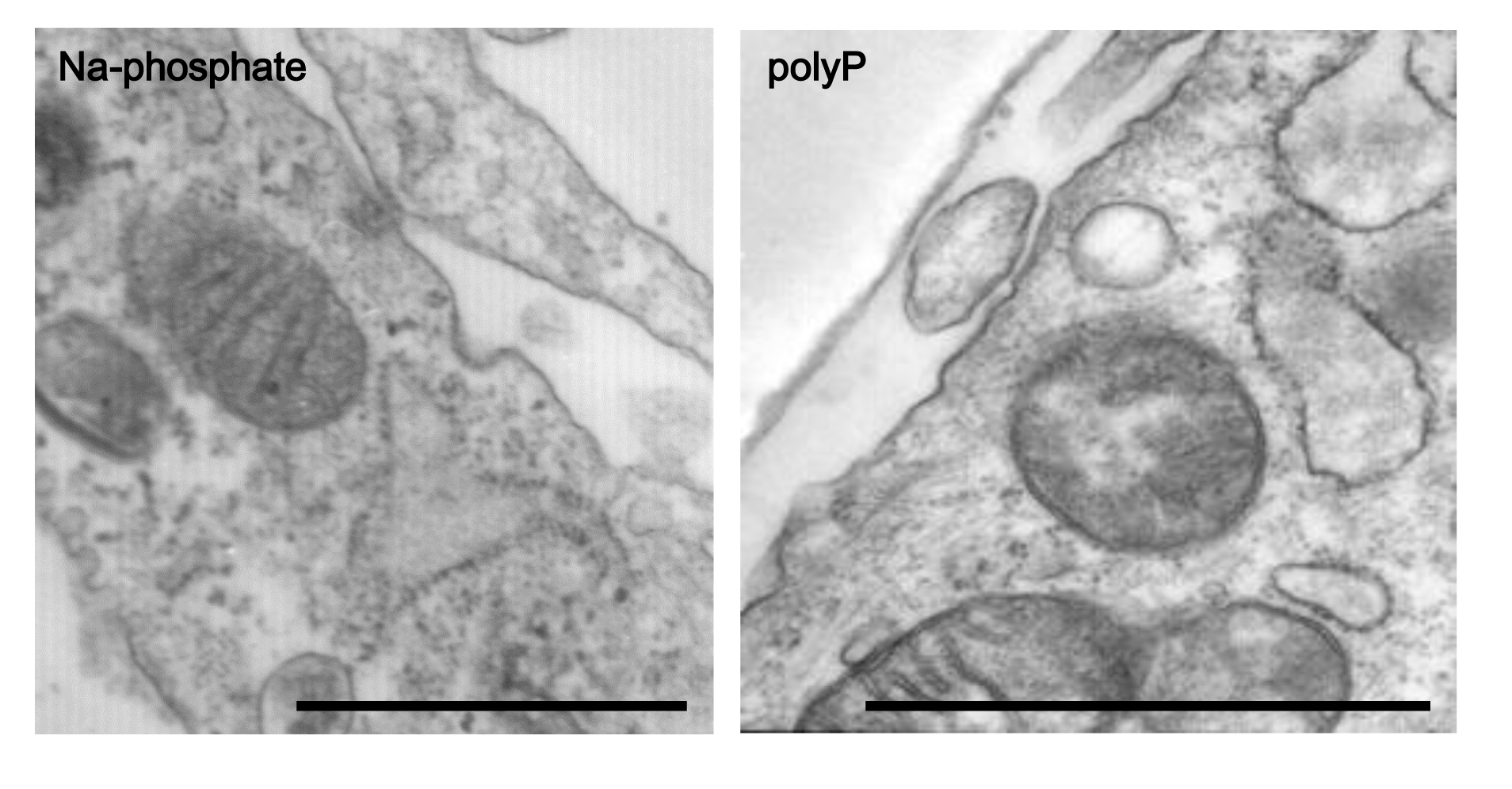

Supplement: Supplementary file 1 — Fig. S1. Electron micrograph of the cells. A. PolyP‐treated cells cultured on Biocoat Control Insert Micron (Becton Dickinson Ltd., USA) were observed using a transmission electron microscope (H‐800; Hitachi, Tokyo, Japan). Scale bar means 1 μm. [file FEB4-9-1617-s001.tif]
